# Early detection of compartment syndrome after aortic dissection with continuous compartment pressure monitoring

**DOI:** 10.1016/j.xjtc.2026.102281

**Published:** 2026-02-14

**Authors:** Joshua G. Crane, Toyokazu Endo, S. Ansley Smith, Jaimin Trivedi, Mark S. Slaughter

**Affiliations:** Department of Cardiovascular and Thoracic Surgery, University of Louisville, Louisville, Ky

**Figure:**
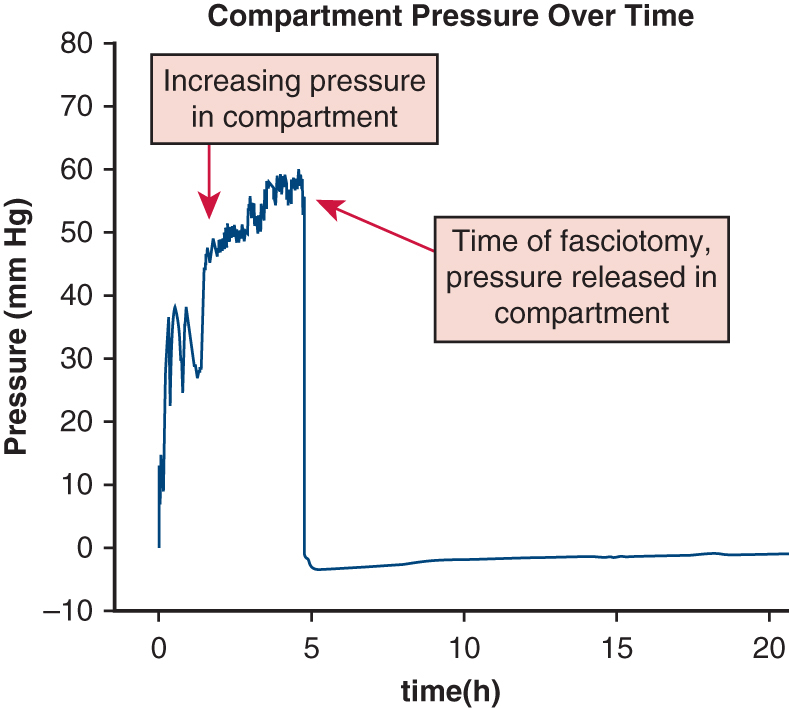
Continuous compartment pressure monitors allow for timely fasciotomy in critical patients.

**Central Message**

Reperfusion-induced compartment syndrome can be identified early with continuous compartment pressure monitors.

Thoracic aortic dissections affect approximately 3 to 4 per 100,000 persons per year.^1^ In patients with a thoracic aortic dissection, extension of the dissection distally can lead to involvement of aortic branches, causing malperfusion syndromes within the kidneys, viscera, and lower extremities. Malperfusion syndromes are present in approximately 70% of these patients, increasing risk of in-hospital morbidity and mortality.^2^ Lower-limb malperfusion is present in approximately 25% of patients with thoracic aortic dissection and is also associated with a greater chance of in-hospital mortality.^2^ Lower-extremity malperfusion may be complicated by reperfusion injury upon recanalization of the native vessels resulting in a compartment syndrome.

Early detection of compartment syndrome is critical to the ability to salvage affected limbs and prevent limb amputation. The MY01 Continuous Compartment Pressure Monitor^3^ has the capability to provide continuous pressure readings in the at-risk extremity. We describe the use of continuous compartment pressure monitoring for early detection of lower-extremity compartment syndrome attributable to reperfusion after a thoracic aortic dissection. Institutional review board approval was received on May 29, 2025 (no. 12.02390), and informed consent was waived.

## Case Report

**Figure 1:**
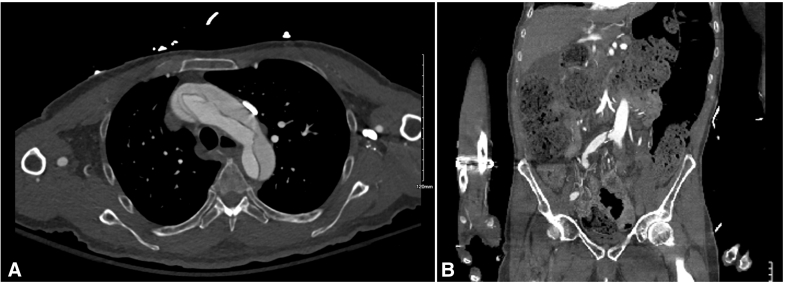
A, Stanford type A thoracic aortic dissection as viewed by computed tomography angiography. B, Left iliac occlusion secondary to the propagated dissection flap.

A 68-year-old man presented to an outside hospital with chest pain, abdominal pain, and lower-extremity numbness. The patient underwent emergent computed tomography angiography of the chest, abdomen, and pelvis, which revealed that the patient had a thoracic aortic dissection beginning in the ascending aorta and extending through the abdominal aorta (Figure 1, *A*). The right iliac artery remained patent to the femoral artery. The left iliac artery was occluded through the popliteal vessels (Figure 1, *B*). The patient was transferred for emergency type A dissection repair.

The patient underwent a standard tube graft replacement of the ascending aorta and hemiarch and resuspension of the aortic valve with moderate hypothermia and antegrade cerebral perfusion. After weaning from cardiopulmonary bypass, there were Doppler signals in the left femoral, posterior tibial, and dorsalis pedis arteries that were not present preoperatively.

**Figure 2:**
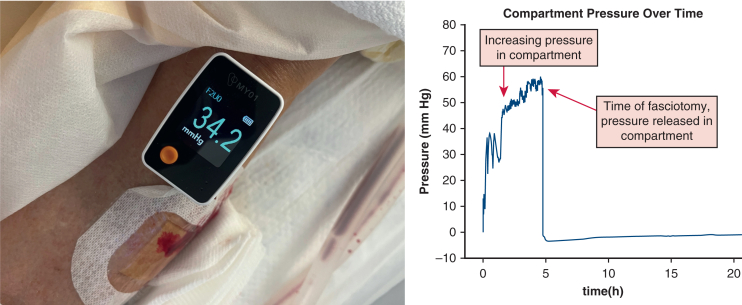
Continuous compartment pressure monitor within the anterior tibial compartment. Monitor readings show elevated pressure and a decrease in pressure with fasciotomy.

Within 1 hour of return to the cardiovascular intensive care unit, despite having palpable pulsatility in the popliteal artery of the left lower extremity as well as posterior tibial artery and dorsalis pedis artery signals in the left foot, the left lower extremity had increased swelling and tightness on examination. A MY01 Continuous Compartment Pressure Monitor was placed within the anterior tibial compartment of the left lower extremity. Within the first hour of placement, continuous pressure monitoring revealed a pressure greater than 30 mm Hg and increasing (Figure 2). Vascular surgery was consulted, and a 4-compartment left lower-extremity fasciotomy revealed bulging, viable muscle in all the released compartments. The patient had negative pressure dressings applied on postoperative day 3 and continued secondary intention closure with vacuum assistance. At discharge, the patient's left lower extremity had no motor or neurologic dysfunction.

## Discussion

Thoracic aortic dissections are associated with a high in-hospital mortality risk.^1^ Mortality risk is complicated further by malperfusion syndromes that are associated with thoracic aortic dissections.^2^ Lower-limb malperfusion syndromes are commonly associated with thoracic aortic dissections.^2^^,^^4^ Secondary to the malperfusion syndrome, reperfusion injury leading to compartment syndrome can follow revascularization.

Classically, compartment syndrome has been considered a clinical diagnosis. Clinicians often are taught to identify compartment syndrome using the “6 Ps”: pain, pallor, pulselessness, paresthesias, paralysis, and poikilothermia. Pallor, poikilothermia, and pulselessness are physical examination signs that may indicate developing compartment syndrome; however, in patients who are hemodynamically unstable and critically ill with comorbidity who are on vasopressors, these signs may be sensitive but not specific for compartment syndrome. In addition, in patients who are critically ill, assessment of pain, paresthesia, and paralysis is not possible, limiting the clinical diagnosis of compartment syndrome.

Application of continuous compartment pressure monitors to extremities at risk from the thoracic aortic dissection allows clinicians to promptly identify limbs at risk in patients who are incapacitated and potentially before the later clinical signs are evident. After placement under ultrasound guidance, the MY01 catheter allows for pressure reading every second for 18 hours. Previous studies have shown the continuous compartment pressure monitors allow for diagnosis of compartment syndrome up to 4 hours earlier than traditional examination, with no false-positive results or missed cases.^5^ The early identification of lower-extremity compartment syndrome spares patients the morbidity associated with limb amputation and may reduce mortality risk associated with lower-extremity malperfusion associated with thoracic aortic dissections.

## Conflict of Interest Statement

The authors reported no conflicts of interest.

The *Journal* policy requires editors and reviewers to disclose conflicts of interest and to decline handling or reviewing manuscripts for which they may have a conflict of interest. The editors and reviewers of this article have no conflicts of interest.
